# SLE and Serum Complement: Causative, Concomitant or Coincidental?

**DOI:** 10.2174/1874312901711010113

**Published:** 2017-09-30

**Authors:** Vaneet Sandhu, Michele Quan

**Affiliations:** 1 Division of Rheumatology, Loma Linda University Medical Center, Loma Linda, CA, USA; 2 Department of Internal Medicine, Arrowhead Regional Medical Center, Colton, CA, USA

**Keywords:** SLE, Systemic Lupus Erythematosus, SLE flares', Complement, CB-CAPS

## Abstract

**Background:**

Systemic Lupus Erythematosus (SLE) is an incurable autoimmune disorder with complement activation playing a key role in the pathogenesis of immune-mediated tissue injury. While quantifying complement to monitor SLE disease activity has been the standard of care since the 1950s, decreased complement levels are not consistently associated with flares.

**Objective:**

We seek to clarify the SLE phenotype in which complement deficiency is causative, concomitant, or coincidental.

**Methods:**

A PUBMED literature review was conducted using key words 'complement,' 'SLE,’ and ‘SLE flares’ in English-only journals from 1972-2017. Relevant clinical studies and review articles were found that examined the measurement of complement levels in SLE, and more specifically, interpretation of low serum complement levels regardless of disease activity.

**Conclusion:**

Complement activation plays a key role in the pathophysiology of SLE and it is recommended to continue monitoring serum levels of C3 and C4 to assess for disease activity. However, it is important to note that decreased serum complement is not consistently associated with disease flares.

It is clinically important to find novel ways to assess disease activity in SLE. Increased serum levels of cell-bound complement activation products may more accurately reflect disease activity than conventional serum C3 and C4 monitoring.

## 1 INTRODUCTION

Systemic Lupus Erythematosus (SLE) is a heterogeneous incurable autoimmune disorder characterized by both B- and T-cell dysfunction that results in immune-mediated multi-system tissue damage. The associations of SLE with end-organ damage, its disproportionate involvement of young females and greater disease burden and severity in minority populations [1] emphasize the importance of timely diagnosis and intervention. The current diagnosis of SLE relies on a combination of history and physical examination findings as well as laboratory criteria [2]. Because of the heterogeneity of the disease and less than ideal sensitivity and specificity of immunologic testing, diagnosing individuals with SLE and then monitoring their disease activity have proven to be difficult.

While standard laboratory testing for SLE includes antinuclear antibody (ANA) and anti-double-stranded DNA (anti-dsDNA) [3] among other autoantibodies, complement activation has proven pivotal in the pathogenesis of SLE-related immune complex damage [4] Fig. (**1**). For years, serum complement proteins, C3 and C4 specifically, have been used to gauge SLE disease activity (SLEDAI); so much so that recent recommendations have included reduced serum complement levels (C3, C4, CH50) in the classification criteria of SLE [2]. However, a confounding factor often encountered in monitoring serum complement levels to ascertain SLE disease activity lies within the subset of SLE patients with a primary complement deficiency. In this subset of patients, who may be asymptomatic, complement levels may indefinitely be low and thus result in misinterpretation of laboratory testing. To this effect, ongoing biomarker research has now also implicated monitoring cell-bound complement activation products (CB-CAPS) in place of serum complements with greater sensitivity in SLE diagnosis [5, 6]. In the present study, we seek to clarify the SLE phenotype in which complement deficiency is causative, concomitant, or coincidental to guide clinicians in providing timely and appropriate management.

## 2 METHODS

We conducted a PUBMED literature review using key words ‘complement,’ ‘SLE,’ and ‘SLE flares in English only journals from 1972-2017. Relevant clinical studies and review articles were found that examined the measurement of serum complement levels in SLE. Of particular interest was the interpretation of low complement levels in patients irrespective of their individual disease activity.

### 2.1 The Complement System and SLE

The complement system is a complex pathway in the immune system composed of proteins and receptors that improve the ability of antibodies and phagocytes to remove microbes and damaged cells from an organism. In addition to its function in combating infectious diseases, it also plays a key role in the inflammatory response prompted by immune complex deposition in numerous autoimmune diseases, including SLE.

Most complement proteins circulate in an inactive form and can be activated by three pathways: the classical pathway, the alternative pathway and the lectin pathway Fig. (**2**). Regardless of the pathway, cleavage of C3 and C5 ultimately occurs, *producing C3a and C5a anaphylatoxins, C3b opsonins, and C5b for the creation of the membrane attack complex* (MAC), which comprises fragments of C3b, iC3b and C3d that act as a bridge to phagocytic cells. There is also the *production of chemotactic factors, inflammatory modulators* [7-9]* and antibody production,* whereby antigens bind to C3d, which interacts with complement receptors and B cell receptors on B-type lymphocytes that *activate, proliferate and produce antibodies* [10]. The complement pathway then assists in clearance of immune complexes and cell lysis *via* the membrane attack complex and removal of apoptotic cells and debris *via* C1q, C4b and C3b, which enhance the ingestion of dead cells by phagocytes [11-13].

Numerous studies have demonstrated a decrease in complement proteins in patients with active SLE, for which quantifying complements have become a standard of care since the 1950s [14-16]. Inherited deficiencies of the complement system, however, demonstrate an independent predisposition of affected individuals to bacterial infections and SLE [17-19]. Complement deficiencies are said to comprise between 1 and 10% of all primary immune deficiencies [18], with an increased association of early complement pathway deficiencies (C1q, C2, C4) in autoimmune diseases [20, 21] like SLE and deficiencies in late complement pathway components (C5-C9) associated with infections, particularly pneumococcal, influenza, and Neisseria infections [22].

It is the loss of self-tolerance that results in autoantibody production in SLE patients with impaired clearance of apoptotic debris. C1q and other proteins typically assist in the removal of apoptotic material, which may explain why C1q deficiency has specifically been associated with impaired immune complex and apoptotic cell clearance [23]. Anti-C1q antibodies also bear a direct association with renal involvement in SLE and potential predilection for renal flares. The central pathway for complement activation in SLE is the classical pathway when activated by immune complexes, which present decreased serum levels of C1q (90-93%), C1r/s (50-57%), C4 (75%), and C2 (10%) (14-17). A more recent review of C1q-deficient patients identified the SLE phenotype to include discoid rash, oral ulcers and anti-smith antibodies with an unusually negative anti-dsDNA and less frequent arthritis [24, 25], implicating potential utility in screening dsDNA-negative cutaneous lupus with serum C1q levels.

While C3 may also be low in SLE, its levels tend to remain normal due to the inhibition of classical pathway activation by C4 binding protein [26]. Low levels of C3 are usually associated with low levels of Factor B, indicating an increased C3 turnover through involvement of the alternative pathway [27, 28].

C4 protein is encoded by two genes (C4A and C4B), each with different functions and different genes on chromosome 6 [29]. Both components demonstrate differences in binding reactivity, with C4B binding twice as effectively as C4A to antibody-coated RBCs but C4A more avidly binding to the protein-antigen complexes. Complete or homozygous deficiency of C4 [missing both C4A genes, reflecting homozygosity for human leukocyte antigen [HLA-B8 (DR3)] is uncommon in the general population but is one of the strongest genetic risk factors for SLE and may result in lupus-like disease in up to 80% of known affected individuals [30-32]. Of note, the most consistent HLA associations with SLE include HLA-DRB1*03:01 (DR3) and HLA-DRB1*15:01 (DR2) and their respective extended haplotypes in European populations [33]. Though rarely done in SLE, genetic testing is required to determine C4 genetic deficiency; levels of C4 may be 0, 25, 50, or 75 percent of normal, depending upon whether the specific individual inherits four, three, two, or one non-expressed allele(s), respectively. In these individuals, the C4 level is at borderline being low throughout life and, if SLE develops, the level of C4 tends to decline more than the baseline levels. Furthermore, because these patients will continue to show low or low-normal total C4, even with effective treatment of disease, our tendency to rely on declining C4 as a surrogate for SLE flares becomes limited. There are two explanations for this picture:

1. The disease is in remission and patient is C4A-deficient (*i.e.* lacks expressed C4A alleles) such that the patient will indefinitely have a low serum C4.

2. There is ongoing classical pathway activation due to active disease (it is important in this scenario to repeat C4 to ensure that the levels are, in fact, low and not due to lab error). As noted, the interpretation of a low serum C4 in a patient with SLE can be complex because reduced levels may be due to the complement consumption or deficiency of one or more alleles or even both scenarios in one patient. In addition, while inherited deficiencies of the complement system are characterized by the static absence of a single complement component, in consumption (as in SLE), there is a fluctuation over time and a decrease in multiple components of complement *i.e*. low C2, C3, and C4. In the face of uncertainty, a blood sample may be sent to a commercial laboratory specializing in complement tests. To determine the number of C4 alleles at the DNA level, the sample would need to be sent to a research laboratory where HLA typing can determine if the patient carries the HLA-B8, DR3 haplotype. Definitive reviews have been published on determining the number of C4 genes in a large number of normal controls and SLE patients [34-36].

The total hemolytic complement (THC or CH50) assesses the ability of serum to lyse sheep red blood cells sensitized with rabbit immunoglobulin M antibody. All components of the classical pathway are required to give a normal CH50, which makes it a useful tool for detecting a deficiency of the classical pathway. It does not assess the alternative pathway because factors B, D, and properdin are not required for the classical pathway. A very low or zero CH50 can result from a genetic deficiency of one or more complement proteins. Moderate reductions in CH50 are often seen in pathologic processes secondary to immune complex formation [37].

While complement activation is a hallmark of the pathogenesis of SLE with decreases associated with hematologic and renal disease [38], decreased complement levels are not consistently associated with flares. In fact, drawbacks of measuring complement levels to assess disease activity include:

1. Variations in complement protein levels due to the individual genetic polymorphisms.

2. Production and breakdown of complement proteins vary with SLE disease activity and individual synthesis response variability. C3 and C4 are also acute phase reactants, implicating an increased synthesis rate in response to inflammatory stimuli. Similarly, this increased synthesis rate may compensate for the increased breakdown demonstrated in SLE due to complement activation [39].

3. Serum complement levels may vary when compared to the tissue levels as demonstrated previously with glomerular basement membrane deposition of complement [40, 41].

4. Autoantibodies to C1q may activate complement in vivo and activate the complement system regardless of disease activity.

Additional studies measuring complement activation products allow for a different, albeit controversial, approach to monitoring SLE disease activity. Elevated levels suggest that SLE (or other diseases in question) is active, while low levels imply that consumption is greater than synthesis [42]. While this is older literature, a functional assay was suggested at the time for all patients with SLE. Unfortunately, direct measurement of complement activation products is not routinely available, is short-lived and may also be affected by activation during the procurement and freeze thawing. Recent data, however, has identified serum cell-bound complement activation products (CB-CAPS) with half-lives as long as the hematopoietic cells with which C3d and C4d (erythrocytes EC4d, B-type lymphocytes BC4d) are bound [43]. These CB-CAPS are identified in most patients with SLE and have demonstrated greater sensitivity and specificity compared to the use of low serum complements for the diagnosis of SLE [6, 44]. Similar studies have demonstrated that elevated levels of complement split products, particularly those of the alternative and terminal pathway activation, may more accurately reflect disease activity than conventional monitoring of complement C3 and C4 in predicting an impending SLE flare [41, 45]. More studies are indicated to clarify this concept and are ongoing with CB-CAPS demonstrated to be quite specific (80-90%) for and present even in mild SLE [43, 44].

The Avise Lupus test was developed in response to the limitations of current diagnostic tests for SLE [6]. It incorporates objective measurement of CB-CAPs and autoantibodies associated with connective tissue diseases. Validation studies have demonstrated that the test has high sensitivity and specificity, and even higher sensitivity than the American College of Rheumatology (ACR) score [6]. This supports earlier findings that CB-CAPs are valuable biomarkers of SLE and that the Avise Lupus test can be an effective supplement in the diagnosis of SLE when clinical and immunological features of SLE are insufficient [46, 47].

## CONCLUSION

The SLE classification criteria was initially set forth in 1982 [48] by the ACR, which was revised in 1997 [49-52]. The Systemic Lupus Collaborating Clinics (SLICC) cohort published a new set of validated criteria in 2012, to include at least one clinical and one immunological criterion in 4 of 17 criteria (Table **1**). One of these criteria includes low serum complement levels of C3, C4 and total complement (CH50), signifying the vital role of complement in SLE.

Testing complement levels has been a standard component of laboratory evaluation to help assess disease activity when monitoring patients with SLE. Low complement levels often signify active lupus, especially lupus nephritis. However, it is difficult to ascertain whether low complement levels are due to consumption during inflammation or due to an inherent deficiency of one or more alleles. Even more obfuscating, the two scenarios may exist in one individual.

Complement activation is an important component of SLE pathogenesis and it is still recommended to continue monitoring serum levels of C3 and C4 to assess for disease activity. However, decreased complement levels are not consistently associated with disease flares, and the disadvantages of measuring complement levels include variations in genetic polymorphisms, synthesis variability and autoantibodies that may activate complement in vivo irrespective of disease activity.

It is clinically important to find novel ways to assess disease activity in SLE. With recent studies demonstrating that increased levels of serum cell-bound complement activation products may more accurately reflect disease activity than conventional complement C3 and C4 monitoring, we believe that this is an important area for future SLE research and look forward to further studies on research in the complement in SLE.

## Figures and Tables

**Fig. (1):**
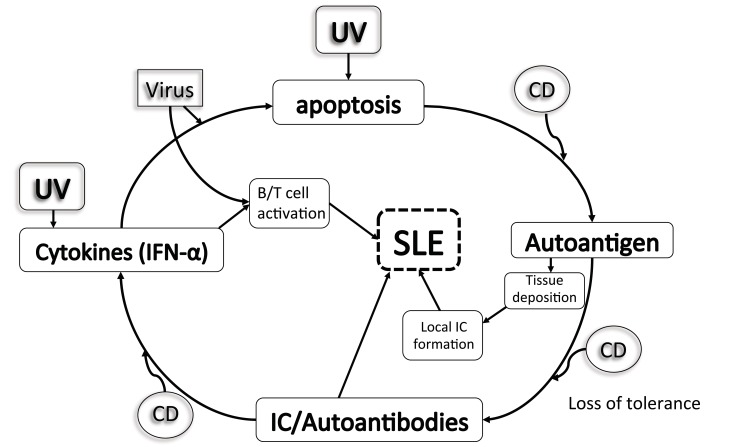
**Suggested roles for complement in the pathogenesis of SLE.** Complement dysfunction (CD) is proposed to accelerate several steps in the pathogenic pathways. Ultraviolet (UV) light and viral infection are examples of environmental influence. IC, immune complex.

**Fig. (2):**
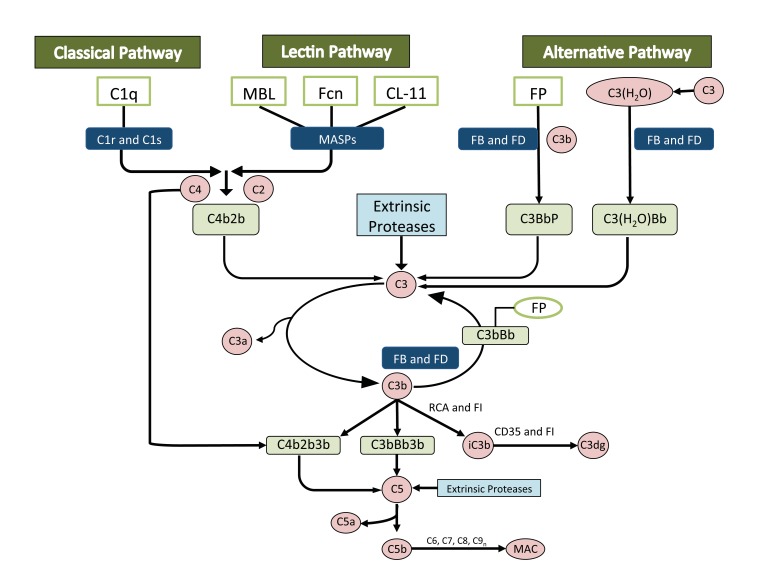
**Complement activation through three distinct ’pathways’: the ‘classical pathway’; the ‘lectin pathway’ and the ‘alternative pathway.’** FB: Factor B, FD: Factor D, FP: Factor P (Properdin), FI: Factor I, RCA: Regulators of Complement Activation, MAC: Membrane Attack Complex

**Table 1:** Classification criteria for systemic lupus erythematosus.

ACR Criteria for Classification of Systemic Lupus Erythematosus [48, 49]		SLICC Criteria for the Classification of Systemic Lupus Erythematous [50]	
(4 of 11 criteria)*		(4 of 17 criteria, including at least one clinical criterion and one immunologic criterion; OR biopsy proven lupus nephritis) Δ	
Criterion	Definition	Criterion	Definition
Clinical Criteria			
Malar Rash	Fixed erythema, flat or raised, over the malar eminences, tending to spare the nasolabial folds	Acute Cutaneous Lupus	Lupus malar rash (do not count if malar discoid); bullous lupus; toxic epidermal necrolysis variant of SLE; maculopapular lupus rash; photosensitive lupus rash (in the absence of dermatomyositis); **OR** subacute cutaneous lupus (nonindurated psoriaform and/or annular polycyclic lesions that resolve without scarring, although occasionally with postinflammatory dyspigmentation or telangiectasias)
Photosensitivity	Skin rash as a result of unusual reaction to sunlight, by patient history or clinician observation	Chronic Cutaneous Lupus	Classic discoid rash; localized (above the neck); generalized (above and below the neck); hypertrophic (verrucous) lupus; lupus panniculitis (profundus); mucosal lupus; lupus erythematosus tumidus; chilblains lupus; OR discoid lupus/lichen planus overlap
Discoid Rash	Erythematosus raised patches with adherent keratotic scaling and follicular plugging; atrophic scarring may occur in older lesions	Nonscarring Alopecia	Diffuse thinning or hair fragility with visible broken hairs (in the absence of other causes, such as alopecia areata, drugs, iron deficiency, and androgenic alopecia)
Oral Ulcers	Oral or nasopharyngeal ulceration, usually painless, observed by a clinician	Oral or Nasal Ulcers	Palate, buccal, tongue, OR nasal ulcers (in the absence of other causes, such as vasculitis, Behçet's disease, infection [herpesvirus], inflammatory bowel disease, reactive arthritis, and acidic foods)
Arthritis	Nonerosive arthritis involving two or more peripheral joints, characterized by tenderness, swelling, or effusion	Joint Disease	Synovitis involving two or more joints, characterized by swelling or effusion OR Tenderness in two or more joints and at least 30 minutes of morning stiffness
Serositis	Pleuritis – Convincing history of pleuritic pain or rubbing heard by a clinician or evidence of pleural effusion OR	Serositis	Typical pleurisy for more than one day, pleural effusions, or pleural rub, OR
	Pericarditis – Documented by ECG, rub, or evidence of pericardial effusion		Typical pericardial pain (pain with recumbency improved by sitting forward) for more than one day, pericardial effusion, pericardial rub, or pericarditis by electrocardiography in the absence of other causes, such as infection, uremia, and Dressler's syndrome
Renal Disorder	Persistent proteinuria greater than 500 mg/24 hours or greater than 3+ if quantitation not performed OR	Renal	Urine protein-to-creatinine ratio (or 24-hour urine protein) representing 500 mg protein/24 hours, OR
	Cellular casts – May be red cell, hemoglobin, granular, tubular, or mixed		Red blood cell casts
Neurologic Disorder	Seizures OR psychosis – In the absence of offending drugs or known metabolic derangements (uremia, ketoacidosis, or electrolyte imbalance)	Neurologic	Seizures; psychosis; mononeuritis multiplex (in the absence of other known causes, such as primary vasculitis); myelitis; peripheral or cranial neuropathy (in the absence of other known causes, such as primary vasculitis, infection, and diabetes mellitus); OR acute confusional state (in the absence of other causes, including toxic/metabolic, uremia, drugs)
Hematologic Disorder	Hemolytic anemia – With reticulocytosis OR Leukopenia – Less than 4000/mm3 total on two or more occasions OR Lymphopenia – Less than 1500/mm3 on two or more occasions OR Thrombocytopenia – Less than 100,000/mm3 (in the absence of offending drugs)	Hemolytic Anemia	Hemolytic anemia
		Leukopenia or Lymphopenia	Leukopenia (<4000/mm3 at least once) (in the absence of other known causes, such as Felty's syndrome, drugs, and portal hypertension), OR
			Lymphopenia (<1000/mm3 at least once) (in the absence of other known causes, such as glucocorticoids, drugs, and infection)
		Thrombocyto-penia	Thrombocytopenia (<100,000/mm3) at least once in the absence of other known causes, such as drugs, portal hypertension, and thrombotic thrombocytopenic purpura
Immunologic Criteria			
ANA	An abnormal titer of ANA by immunofluorescence or an equivalent assay at any point in time and in the absence of drugs known to be associated with “drug-induced lupus” syndrome	ANA	ANA level above laboratory reference range
Immunologic Disorders	Anti-DNA – Antibody to native DNA in abnormal titer OR Anti-Sm – Presence of antibody to Sm nuclear antigen OR Positive finding of antiphospholipid antibody based on an abnormal serum level of IgG or IgM anticardiolipin antibodies, on a positive test result for lupus anticoagulant using a standard method, or on a false-positive serologic test for syphilis known to be positive for at least six months and confirmed by Treponema pallidum immobilization or fluorescent treponemal antibody absorption test	Anti-dsDNA	Anti-dsDNA antibody level above laboratory reference range (or >twofold the reference range if tested by ELISA)
		Anti-Sm	Presence of antibody to Sm nuclear antigen
		Antiphospholipid	Antiphospholipid antibody positivity as determined by any of the following: Positive test result for lupus anticoagulant; false-positive test result for rapid plasma reagin; medium- or high-titer anticardiolipin antibody level (IgA, IgG, or IgM); or positive test result for anti-beta 2-glycoprotein I (IgA, IgG, or IgM)
		Low Complement	Low C3; low C4; OR low CH50
		Direct Coombs’ Test	Direct Coombs' test in the absence of hemolytic anemia

ACR: American College of Rheumatology; SLICC: Systemic Lupus International Collaborating Clinics; SLE: systemic lupus erythematosus; ECG: electrocardiogram; ANA: antinuclear antibodies; Anti-Sm: anti-Smith antibody; IgG: immunoglobulin G; IgM: immunoglobulin M; Anti-dsDNA: anti-double-stranded DNA; ELISA: enzyme-linked immunosorbent assay; IgA: immunoglobulin A. * For the ACR criteria, no distinction is made between clinical and immunologic criteria in determining whether the required number has been met. The classification is based upon 11 criteria. For the purpose of identifying patients in clinical studies, a person is said to have SLE if any 4 or more of the 11 criteria are present, serially or simultaneously, during any interval of observation. ¶ For the SLICC criteria, criteria are cumulative and need not be presently concurrently. A patient is classified as having SLE if he or she satisfies four of the clinical and immunologic criteria used in the SLICC classification criteria, including at least one clinical criterion and one immunologic criterion. Δ Alternatively, according to the SLICC criteria, a patient is classified as having SLE if he or she has biopsy-proven nephritis compatible with SLE in the presence of ANAs or anti-dsDNA antibodies.
